# The relationship between cyberchondria and health literacy among first-year nursing students: the mediating effect of health anxiety

**DOI:** 10.1186/s12912-024-02396-9

**Published:** 2024-10-22

**Authors:** Samira Saad Ali, Nourhan Essam Hendawi, Ayman Mohamed El-Ashry, Manal Saeed Mohammed

**Affiliations:** 1 Medical-Surgical Nursing Department, Faculty of Nursing, Alexandria University, Alexandria, Egypt; 2 Nursing Education Department, Faculty of Nursing, Alexandria University, Alexandria, Egypt; 3 Psychiatric and Mental Health Nursing Department, Faculty of Nursing, Alexandria University, Alexandria, Egypt; 4 Psychiatric and Mental Health Nursing, Department, of Nursing, College of Applied Medical Sciences, Jouf University, Al-Qurayyat, Saudi Arabia; 5 Medical Surgical Nursing Department, Faculty of Nursing, Alexandria University, Alexandria, Egypt

**Keywords:** Cyberchondria, Health literacy, Hypochondriasis, Chronic disease, Nursing students, Health anxiety

## Abstract

**Background:**

First-year nursing students are at a critical juncture in their education. They are transitioning from laypersons to healthcare professionals, and students are frequently exposed to medical information in academic settings and through personal research. This exposure can sometimes lead to cyberchondria; improving health literacy and managing health anxiety are critical strategies for reducing the incidence of cyberchondria.

**Aim:**

Investigate the mediating effect of health anxiety on cyberchondria and health literacy among first-year nursing students.

**Methods:**

A cross-sectional correlational research design was used in this study. The study was conducted with 475 students in October 2023 during the first semester of the academic year 2023–2024.

**Results:**

Cyberchondria and health literacy levels were both moderate. In addition, the degree of health anxiety among first-year Nursing Students was mild, too. The results showed that the association between Cyberchondria and Health Literacy was partially mediated by health anxiety.

**Conclusion and implications:**

This study highlights the sophisticated relationship between cyberchondria, health literacy, and anxiety among first-year nursing students. It demonstrates that lower health literacy can lead to increased health anxiety, which in turn exacerbates cyberchondria. To mitigate these issues, it is essential to enhance health literacy and provide support for managing health anxiety within nursing education programs. By doing so, we can help nursing students navigate online health information effectively and reduce unnecessary health-related anxieties, promoting better educational outcomes and overall well-being.

## Introduction

 First-year nursing students are at a pivotal stage in their education as they shift from everyday individuals to healthcare professionals. During this transition, they are often inundated with medical knowledge through coursework and personal research [1, 2]. This constant exposure to medical information can sometimes result in Cyberchondria, where students may develop heightened anxiety about their health. Enhancing health literacy and effectively managing health-related anxiety are crucial strategies to help reduce the risk of Cyberchondria among these students [3, 4].

Cyberchondria is a relatively new term used to describe excessive or repeated online searching for health-related information, often leading individuals to believe they are suffering from severe medical conditions. While the Internet can be a helpful resource for learning about health issues, it can also have unintended adverse effects, particularly for those predisposed to health anxiety or hypochondria [4]. This behavior often results in heightened distress, as people misinterpret common or minor symptoms as indicators of severe illnesses [5]. According to Starcevic et al. (2020), a national survey of American adults revealed that 35% of participants had self-diagnosed a medical condition based on online searches within the past year [6].

Nursing students, in particular, are at risk of developing Cyberchondria. They may be more prone to self-diagnosis as they acquire valuable and diverse knowledge about severe diseases and conditions through their education [7]. This tendency is exacerbated by their frequent use of the Internet to seek health information, both during formal education and independently. As a result, these students may perceive themselves as having severe illnesses or fear developing them, which leads to increased health anxiety [7, 8]. Research has shown that individuals with high levels of health anxiety are more likely to search for health-related information online, which can worsen their anxiety and contribute to Cyberchondria [4, 5].

Cyberchondria can have a significant impact on nursing students. It often leads to elevated levels of anxiety and stress and, in some cases, hypochondria. This can affect their ability to focus on their studies and perform effectively in clinical settings. Relying on online sources for self-diagnosis also increases the risk of misinformation, potentially leading to harmful self-treatment practices. Therefore, nursing students must learn to critically assess online health information and seek advice from healthcare professionals when necessary [9, 10].

Nursing programs are already known to be rigorous, placing significant psychological demands on students, which can affect their physical and emotional well-being and academic success. Since nursing students are frequently exposed to critical diseases through their studies and practical experiences, they are more likely to develop health-related anxiety. This is sometimes referred to as hypochondriasis, nursing students’ syndrome, or simply health anxiety [11]. In light of these risks, nursing schools must provide students with the tools to manage health-related stress and anxiety effectively.

Health literacy is crucial in how individuals engage with online health information. It can empower individuals to make informed health decisions or leave them susceptible to Cyberchondria if they struggle to effectively evaluate the information encountered [12]. Therefore, promoting and enhancing Health literacy skills among individuals, including nursing students, is essential in mitigating the negative impacts of Cyberchondria and fostering healthier online health information behaviors. When individuals can effectively evaluate the credibility and relevance of online health information, they are less likely to succumb to excessive health anxiety or unnecessary worry based on their online searches [12].

The capacity to comprehend and apply health information to make well-informed decisions about one’s health is known as health literacy. It is a collection of personal competencies that enable individuals to obtain and utilize knowledge for self-care and healthcare decision-making [13]. Thus, health literacy includes knowing and assessing the adequacy of health-related information and making self-care and illness management decisions. It also involves using health services, drugs, and informed consent forms appropriately. On the other hand, low health literacy can result in a rise in drug abuse, unhealthy habits, inadequate disease management, and limited access to care services. These factors can eventually raise the risk of illness and death, worsen personal quality of life, and raise societal expenses [14].

Health anxiety is a psychiatric condition marked by intense anxiety in which students mistakenly connect the nebulous symptoms they experience to the symptoms of a specific disease they are studying, leading to a dread of having that sickness. Furthermore, based on their current clinical rotation, the students may revise the diagnosis of their disease. Sadly, these anxieties continue even after receiving medical assurance, which stresses the students out and impairs their ability to focus throughout training [15].

Many factors can affect a student’s health literacy, including age, gender, socioeconomic status, and parent education level. However, one of the most important factors is the quality of health education they receive in university. A study by the World Health Organization found that students who received high-quality health education had better health literacy skills than those who did not. The study also found that health education can help prevent chronic diseases, improve mental health, reduce risky behaviors, and reduce the unwanted effects of Cyberchondria [16].

A growing body of research suggests a relationship between Cyberchondria, health anxiety, and low health literacy among nursing students. Nursing students who have low health literacy may be more likely to rely on the Internet for health information, which can lead to anxiety and confusion about their health [17]. Nursing students need to develop robust health literacy skills to effectively navigate the vast amount of online health information and make informed decisions about their health. This can be achieved through education and training programs that focus on improving health literacy skills and providing nursing students with the tools needed to critically evaluate health information found online [18].

### Significance of the study

While there is extensive research on Cyberchondria (the tendency to excessively search for medical information online and develop health anxiety), there is limited focus on its relationship with health literacy, particularly among nursing students. First-year nursing students are a unique population in this context due to their growing exposure to medical knowledge. While they are expected to develop critical thinking skills regarding health information, their inexperience may make them vulnerable to the negative effects of Cyberchondria, exacerbated by health anxiety. The current literature lacks specific insights into how health literacy influences Cyberchondria in this group and, more importantly, how health anxiety mediates this relationship. Addressing this gap is essential to designing effective educational strategies that not only enhance health literacy but also mitigate health anxiety and its associated risks in future healthcare professionals.

### Aim of the study

- Investigate the mediating effect of health anxiety between Cyberchondria and Health Literacy among first year Nursing Students.

### Research question

- How health anxiety mediates the relationship between Cyberchondria and Health Literacy among first year Nursing Students?

### Design and participants

This study used a cross-sectional correlational research design. The questionnaire was chosen to collect data on students’ health information-seeking behaviors, their levels of health literacy, and their experiences with health anxiety at a single time. The study was conducted in October 2023, during the first semester of the academic year 2023–2024.

### Participants

A convenient sample size of all nursing students enrolled at the first academic level at the Faculty of Nursing, Alexandria University, during the first term of the academic year 2023/2024, was recruited to conduct a study. The study included approximately 475 nursing students who fulfilled the following criteria from 520 nursing students, with a response rate of 91.3%.

### Inclusion criteria

Students of both sexes, age 18 years or older, enrolled as first-year nursing students and registered in the Management Information System (MIS) academic program for the academic year 2023/2024, and willing to provide informed consent to participate in the study.

### Exclusion criteria

Nursing students beyond their first year of study. Furthermore, Individuals who did not provide consent or could not complete the survey due to language barriers or other reasons.

### Settings

The study will be conducted at the Medical Surgical Nursing department at the Faculty of Nursing for first-year students at Alexandria University in Egypt.

### Study instruments

The following instruments were used to collect data on cyberchondria, health literacy, and health anxiety among first-year nursing students:

In addition to gathering Students’ socio-demographic characteristics and lifestyle, it included items related to their age, gender, residence, academic year, grade of last semester, chronic disease, self-medication, smoking condition, perceived health, and internet use regarding health. Three tools were used to collect the necessary data about the study variables.

### The cyberchondria severity scale short-form (CSS-12)

The tool was developed by McElroy et al. [19] to assess the severity of cyberchondria in nursing students. This tool consists of 12 questions that will be answered by the students using a 5-point Likert scale. The score was categorized as mild, moderate, and severe cyberchondria based on the total sum of points (0–60). The subjects were also classified based on their score percentage, with 33.3% or less being mild, 33.3% to less than 66.7% being moderate, and more than 66.7% being severe cyberchondria.

### Nursing students’ health-literacy questionnaire

The tool was developed by Nolasco et al. [20] to assess health literacy levels and was adapted by [21] to assess students’ health literacy levels. The scale contains 16 items with a 4-point Likert scale. The responses were scored and categorized into “very difficult or difficult” =0 and “easy or very easy” =1. Based on their scores, the subjects can be classified as inadequate, problematic, and sufficient health literacy. The subjects were classified as inadequate health literacy (1–8 points), problematic health literacy (9–12 points), and sufficient health literacy (13–16 points). The score was converted into percent and categorized as follows: A 48–64% score was considered enough health literacy, a 32–47% score was regarded as problematic health literacy and a 16–31% score was considered inadequate health literacy.

### Whiteley Index (WI) self-reports questionnaire

A 14-item version of the Whiteley Index (WI) was developed by Pilowsky to diagnose hypochondriasis or health anxiety [22]. It consists of 14 items, each of which may be answered with a simple “Yes” or “No” response. There are two types of reactions to these things. “Is it hard for you to believe the doctor when he tells you there is nothing to worry about?” is an example of an item. Except for one item, where the responses were coded as 1 for “Yes” and 0 for “No”—that is, “Yes” received a score of 0, and “No” received a score of 1. Therefore, the total score is calculated by adding the scores of all the components and goes from 0 to 14 [23].

### Procedure

#### Ethical considerations

The study received ethical approval from the Research Ethics Committee (REC) at Alexandria University College of Nursing (IRB00013620/9/2023). Furthermore, an official letter was obtained from the Faculty of Nursing at Alexandria University to secure their approval for data collection after explaining the study’s objectives. All participants provided written informed consent and willingly agreed to participate in the survey. The research team followed strict protocols to maintain the confidentiality of participants. Only the study team had access to the personal data collected, ensuring the privacy and anonymity of participants. Additionally, each participant was informed of their right to withdraw from the study at any time.

#### Pilot study

A pilot research study, including 10% (45) of nursing students, was carried out to evaluate the tools’ clarity and usefulness. The researchers used Cronbach’s alpha test to examine the reliability of the study instruments since they were employed in their English translation. Subsequently, the Whiteley Index (WI) self-report questionnaire, the Cyberchondria Severity Scale Short-Form (CSS-12), and the health-literacy Questionnaire of nursing students yielded significant results on the reliability test (0.88, 0.87, 0.91). The study did not include nursing students who participated in the pilot trial.

#### Data collection

Students were introduced to the researcher, an explanation of the study’s purpose, and an assurance that their answers would remain confidential. All nursing students received a questionnaire after the clinical day to avoid interfering with their learning. They were instructed to complete it and send it back to the researcher. The researcher provided identical instructions to every nursing student upon completing the Questionnaire. Additionally, they said that each question should only have one response and that no question should go unanswered. The researchers addressed any queries posed by the students. It took each student fifteen to thirty minutes to finish the study materials.

#### Statistical analysis of data

Following collection, the data were coded and prepared for computer entry. To avoid input mistakes, thorough checks and verifications were conducted after the data was entered. Several methods were used to find and fix inconsistencies, including frequency analysis, cross-tabulation, and manual review. The Statistical Package for the Social Sciences (SPSS version 25) was used for statistical analysis, including descriptive statistics like numbers, percentages, and averages. The Sobel test, Pearson correlation, and regression analysis were also used as statistical tests. For the study, a *p*-value of 0.05 or less was selected as the significance threshold.

## Results

 Table 1 shows the distribution of the studied students according to their essential characteristics. The table shows that the majority of the students are female (82.5%), aged between 21 and 23, in their first academic year (74.7%), with GPAs almost evenly split between less than 3 and 3 or higher. More students live in urban areas (60.2%) than rural ones.

**Table 1:** Distribution of the studied students according to their basic characteristics

Students’ characteristics	Total *N*=475	
	No.	%
**Sex**		
Male	83	17.5
Female	392	82.5
**Age (years)**		
19-	13	2.7
21-	172	36.2
23-	193	40.6
≥25	97	20.4
**Academic Year**		
First	355	74.7
Second	93	19.6
Third	17	3.6
Fourth	10	2.1
**Last GPA**		
<3	252	53.1
≥3	223	46.9
**Place of residence**		
Rural	189	39.8
Urban	286	60.2

 Table 2 reveals that most students do not have chronic diseases (83.8%) and perceive their general health as good (92.4%). A significant number of students do not practice self-medication (69.9%) and do not use tobacco (85.5%). When facing health problems, most students prefer to visit a doctor (52.0%).

**Table 2:** Distribution of the studied students according to their health-related data

Items	Total *N*=475	
	No.	%
**Have chronic diseases**		
No	398	83.8
Yes	77	16.2
**Perceived general health**		
Poor	36	7.6
Good	439	92.4
**Self-medication**		
No	332	69.9
Yes	143	30.1
**Tobacco use**		
No	406	85.5
Yes	69	14.5
**Think have problems with health**		
No	303	63.8
Yes	172	36.2
**Measures done in case of health problems**		
Wait for recovery	87	18.3
Apply my knowledge	60	12.6
Look for source of problem on internet	63	13.3
Visit a doctor	247	52.0
Consult a friend	18	3.8

 Table 3 presents distributions of the studied students according to their internet use-related data. Most students access the internet via mobile phones (85.3%), use Google (68.4%), and spend 3–5 h daily online. Most students believe the internet is as good as a physician in health information (65.3%). They search the internet for health information both without and after physician consultations. The main topic for online search is diet (35.2%), and they scan health information online less than once per month (43.2%). Most students need clarification about the reliability of health information searched online (58.3%).

**Table 3:** Distribution of the studied students according to their internet use-related data

Items	Total *N*=475	
	No.	%
**Internet access point**		
Computer	41	8.6
Tablet	29	6.1
Mobile phone	405	85.3
**Application used on internet**		
Twitter	43	9.1
Google	325	68.4
YouTube	107	22.5
**Duration of daily internet use (hours)**		
1-	117	24.6
3-	168	35.4
5-	107	22.5
≥7	83	17.5
**Believe internet as good as physician in health information**		
No	165	34.7
Yes	310	65.3
**Search internet about health without physician consultation**		
No	257	54.1
Yes	218	45.9
**Search internet about health after physician consultation**		
No	116	24.4
Yes	359	75.6
**Main topic for search online**		
Disease	148	31.2
Treatment	121	25.5
Diet	167	35.2
Risk behaviors (smoking- alcohol.)	39	8.2
**Frequency of health scan online**		
Less than once per month	205	43.2
Once per month	143	30.1
Once per week	76	16.0
Two times and more per week	51	10.7
**Reliability of health information searched via internet**		
Incorrect	86	18.1
Not sure	277	58.3
Correct	112	23.6

 Table 4 presents the distribution of the studied students according to the Cyberchondria, Health Literacy, and Health Anxiety levels. Most students have moderate Cyberchondria (57.5%), inadequate Health Literacy (46.1%), and experience Health Anxiety (65.5%). The association matrix between health anxiety, health literacy, and Cyberchondria is displayed in Table 5. According to the correlation matrix, Cyberchondria and health literacy have a strong negative link (*r*=-0.529), whereas Cyberchondria and health anxiety have a strong positive correlation (*r* = 0.836). Additionally, there is a strong inverse relationship (*r*=-0.661) between health anxiety and health literacy. At *p* < 0.05, every association is significant. This implies that health anxiety rises and health literacy falls as Cyberchondria rises. In Cyberchondria, in a similar vein, health anxiety declines with increased health literacy.

**Table 4:** Distribution of the studied students according to the levels of Cyberchondria, Health Literacy, and Health Anxiety

Items	Total *N*=475	
	No.	%
**Cyberchondria**		
Low	181	38.1
Moderate	273	57.5
High	21	4.4
**Health Literacy**		
Inadequate	219	46.1
Problematic	101	21.3
Sufficient	155	32.6
**Health Anxiety**		
No anxiety	164	34.5
Anxiety	311	65.5

**Table 5:** Correlation Matrix between Cyberchondria, Health Literacy, and Health Anxiety

		**Cyberchondria**	**Health Literacy**
**Cyberchondria**	r		
	p		
**Health Literacy**	r	-0.529	
	p	0.000*	
**Health Anxiety**	r	0.836	-0.661
	P	0.000*	0.000*

*R* Pearson Correlation

r ≥0.9 very high correlation

r 0.7-<0.9 high correlation

r 0.5-<0.7 moderate correlation

r < 0.5 low correlation

*Correlation is significant at *p* ≤ 0.05

Table 6 showed that health literacy had a positive effect on the health anxiety of first-year nursing students (B = -2.642, *p* < 0.0000), health anxiety had a direct negative effect on the Cyberchondria for nursing students (B = 0.270, *p* < 0.0000). cyberchondria negatively influenced their health literacy (B = 0.662, *p* < 0.0000*), supporting Hypothesis. Thus, health anxiety and health literacy were predictors of Cyberchondria for nursing students. In addition, the table shows that the total effect of health literacy on Cyberchondria was significant (β = -0.266, *p* < 0.0001). Health literacy had a significant indirect impact on Cyberchondria mediated by the role of health anxiety (B = -0.2068, 95% CI = 0.355 to -0.163), accounting for 77.45% of the total effect. Hence, health anxiety partially mediated the relationship between Cyberchondria and health literacy.

**Table 6:** Mediating role of health anxiety in the relation between cyberchondria and health literacy, (*n*=475)

Dependent variables		Independent variable	B	R-sq	SE	95%confidence interval		T	*P* value	Effect proportion
						LL	UL			
**ModelPath**	**Health anxiety**	**Health literacy**	-2.642	0. 0.437	0.213	-0.359	-0.879	12.391	0.000	-
	**Cyberchondria**	**Health anxiety**	0.270	0.700	0.016	0.542	0.783	16.630	0.000	-
	**Cyberchondria**	**Health literacy**	0.662	0.280	0.075	0.115	0.409	8.774	0.000*	21.23%
**Type of effect**	**Direct effect**									
	**Indirect effect**		-2068		0.0235	0.355	-0.163	-	(sig)	77.45%
	**Total effect**		-2660		-0.0330	-0.326	0.0206	-8.734	≤ 0.001*	-

^*^Correlation is significant at *p* ≤ 0.05

*B* Unstandardized regression coefficient, *SE* Standard error, *LL* Lower limit, *UL* Upper limit

**Fig. 1:**
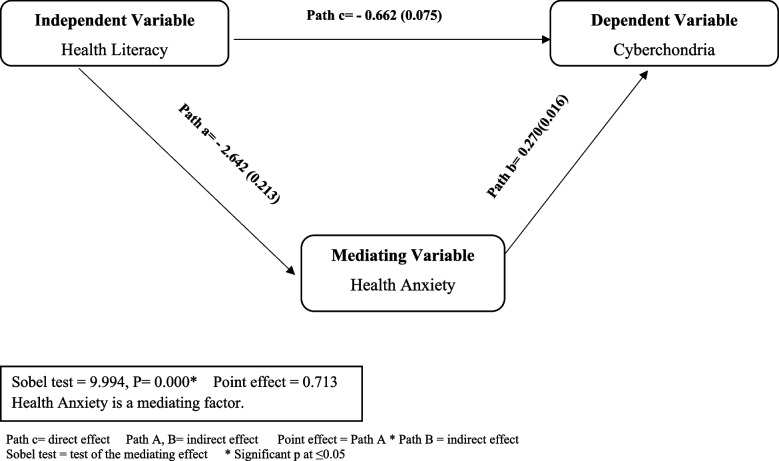
The mediating effect of health anxiety between health literacy and cyberchondria

 Figure 1 shows that Path C (Direct Effect) was the effect of health literacy on the cyberchondia, which was slightly significant (Path c = − 0.662 (0.075). Path A and Path B (Indirect Effect): These paths represent the effect of health literacy on cyberchondia through health anxiety. Path A is the effect of health literacy on health anxiety, and Path B is the effect of health anxiety on Cyberchondria. Point Effect was the product of Path A and Path B, representing the indirect effect of health literacy on Cyberchondria through health anxiety (Point effect = 0.713). Sobel Test shows that health anxiety mediated the relationship between health literacy and Cyberchondria with a statistically significant test (Sobel test = 9.994, *P* = 0.000).

## Discussion

The study’s findings underscore the significant interplay between cyberchondria, health literacy, and health anxiety among nursing students. This revelation that most students exhibit moderate levels of cyberchondria and inadequate health literacy, with a majority also experiencing health anxiety, is a crucial insight. The strong negative correlation between cyberchondria and health literacy, and the positive correlation between cyberchondria and health anxiety, provide a deeper understanding of these phenomena. The model path analysis further confirms that health literacy negatively influences health anxiety, which in turn affects cyberchondria. This supports the hypothesis that health anxiety and health literacy are critical predictors of cyberchondria in nursing students. The indirect effects further demonstrate that health anxiety mediates the relationship between health literacy and cyberchondria, shedding light on the complex dynamics at play.

The majority of students, as indicated by El-Zoghby 2024 [24], primarily use their mobile phones to access the internet, use Google, and spend approximately 3–5 h daily online. These researchers found that most students visited websites like Facebook, WhatsApp, YouTube, and Google. Moreover, students frequently sought health-related information for personal reasons rather than academic purposes, expressing regular concerns about their health and that of their family members. Furthermore, Molu et al. 2023 [25] demonstrated that most participants accessed the internet through their mobile devices, spending 5–6 h online daily.

In this current investigation, it was observed that most students exhibit moderate levels of cyberchondria, possess insufficient health literacy, and manifest health anxiety. This aligns with the findings of those who reported moderate scores on the cyberchondria severity scale [26]. Moreover, El-Zoghby [24] identified a positive correlation between smartphone addiction and moderate-to-high cyberchondria levels. Furthermore, Rogala and Nestorowicz [27] discovered that individuals with higher e-literacy displayed lower cyberchondria levels. Furthermore, Kobryn and Duplaga [28] pointed out that younger individuals tended to have higher cyberchondria scores than older individuals, with men scoring lower than women. Increased health anxiety was significantly linked to cyberchondria severity.

Several factors contribute to cyberchondria, such as nursing students having greater access to health information resources, younger individuals conducting more online health searches and exhibiting higher cyberchondria levels, and individuals with perceived undiagnosed illnesses experiencing heightened health anxiety, increasing cyberchondria behaviors [10]. Consequently, elevated levels of cyberchondria can lead to mental health issues like anxiety, depression, stress, and obsessive-compulsive disorders [10].

Furthermore, the outcomes of experiencing health anxiety may stem from the gender makeup of our sample being predominantly female, given that existing literature highlights the heightened levels of health anxiety in women. This heightened health anxiety may prompt individuals to turn to the internet in search of reassuring information. Nevertheless, the information they come across may fuel further concerns [29].

Recent research has shown that there is a notable positive link between health anxiety and cyberchondria and a substantial negative correlation between cyberchondria and health literacy. Furthermore, there is a noteworthy inverse relationship between health anxiety and health literacy. These findings align with Aydan et al. [30], which indicates that there may not be a statistically significant connection between cyberchondria and e-health literacy. Furthermore, TabeBordbar et al. [31] have also established a noteworthy and affirmative correlation between health worry and cyberchondria. Mert & Keklik 2023 [32] also discovered no discernible relationship between health literacy and health anxiety. Additionally, a strong inverse relationship between health anxiety and health literacy has been observed by Dadgarinejad et al. [33]

The total effect of health literacy on cyberchondria was significant, indicating that health literacy directly reduces cyberchondria. Additionally, the indirect effect of health literacy on cyberchondria, mediated by health anxiety, was substantial, accounting for 77.45% of the total effect. This highlights the crucial role of health anxiety as a mediator in this relationship. The Sobel test further confirmed the mediating role of health anxiety, emphasizing that health anxiety partially mediates the relationship between health literacy and cyberchondria.

This was evidenced by a study done by Bajcar and Babiak (2021), which found that self-esteem directly predicted cyberchondria and that health anxiety and obsessive-compulsive symptoms mediated this relationship. This means that individuals with lower self-esteem are more likely to experience cyberchondria, and this effect is partly due to increased health anxiety and obsessive-compulsive behaviors [34].

### Limitations and recommendations

Given the increasing reliance on the internet for health information, the study reports a timely and pertinent issue, making its findings highly relevant to ongoing nursing education and practice. The study’s findings not only enhance our understanding of these issues but also inspire action. In addition to focusing on first-year nursing students, the study provides valuable insights into the early stages of their professional development, a critical period for shaping their information-seeking behaviors and attitudes toward health information. This study has a few limitations, but it also opens up new avenues for research and intervention, motivating future studies to build on these findings.

Further, longitudinal studies are advised to establish causal relationships. Finally, other factors such as prior healthcare experience, personality traits, mental health status, and existing health conditions were not controlled, which might influence the outcomes. Future studies should consider additional variables to better understand their influence on the relationship between health literacy, health anxiety, and cyberchondria.

## Conclusions

In conclusion, the study highlights that health literacy and health anxiety significantly influence Cyberchondria among nursing students. As Cyberchondria increases, health literacy decreases, and health anxiety rises. Health anxiety partially mediates the relationship between health literacy and Cyberchondria, with lower health literacy leading to higher health anxiety, which in turn exacerbates Cyberchondria. This underscores the importance of improving health literacy to reduce health anxiety and mitigate the impact of Cyberchondria, especially among healthcare students.

### Implications of the study

The findings in the current study underscore the importance of addressing health anxiety and improving health literacy to mitigate cyberchondria among nursing students. This not only enhances our understanding of these issues but also provides actionable insights. Enhancing health literacy could reduce health anxiety, decreasing the tendency towards cyberchondria. This cyclical relationship suggests that interventions aimed at improving health literacy and managing health anxiety could be effective strategies for reducing cyberchondria, empowering nursing educators, healthcare professionals, researchers, and policymakers with practical solutions.

Nursing management should integrate comprehensive health literacy training into the curriculum. This training should focus on developing critical appraisal skills for evaluating online health information, distinguishing between reliable and unreliable sources, and applying this knowledge in clinical practice. In addition, training faculty members to recognize signs of cyberchondria and health anxiety in students can enable early intervention. Faculty can be prepared with strategies to support students in managing their anxieties and improving their health literacy. By implementing these strategies, nursing management can help create an educational environment that supports the development of high health literacy, reduces health anxiety, and minimizes cyberchondria among nursing students. This will improve students’ academic experiences and prepare them to be more competent and confident healthcare professionals.

## Data Availability

The datasets used and/or analysed during the current study are available from the corresponding author on reasonable request.

## Abbreviations

WI: Whiteley Index
CSS-12: Cyberchondria Severity Scale Short-Form 12
MIS: Management Information System
SPSS version 25: The Statistical Package for the Social Sciences
